# Detection and specific chemical identification of submillimeter plastic fragments in complex matrices such as compost

**DOI:** 10.1038/s41598-024-51185-6

**Published:** 2024-01-27

**Authors:** Thomas Steiner, Lisa-Cathrin Leitner, Yuanhu Zhang, Julia N. Möller, Martin G. J. Löder, Andreas Greiner, Christian Laforsch, Ruth Freitag

**Affiliations:** 1 Process Biotechnology, University of Bayreuth, Universitätsstrasse 30, 95440 Bayreuth, Germany; 2 Macromolecular Chemistry II, University of Bayreuth, Bayreuth, Germany; 3 Animal Ecology I & BayCEER, University of Bayreuth, Bayreuth, Germany

**Keywords:** Environmental sciences, Environmental impact, Ecology

## Abstract

Research on the plastic contamination of organic fertilizer (compost) has largely concentrated on particles and fragments > 1 mm. Small, submillimeter microplastic particles may be more hazardous to the environment. However, research on their presence in composts has been impeded by the difficulty to univocally identify small plastic particles in such complex matrices. Here a method is proposed for the analysis of particles between 0.01 and 1.0 mm according to number, size, and polymer type in compost. As a first demonstration of its potential, the method is used to determine large and small microplastic in composts from eight municipal compost producing plants: three simple biowaste composters, four plants processing greenery and cuttings and one two-stage biowaste digester-composter. While polyethylene, PE, tends to dominate among fragments > 1 mm, the microplastic fraction contained more polypropylene, PP. Whereas the contamination with PE/PP microplastic was similar over the investigated composts, only composts prepared from biowaste contained microplastic with a signature of biodegradable plastic, namely poly(butylene adipate co-terephthalate), PBAT. Moreover, in these composts PBAT microplastic tended to form the largest fraction. When the bulk of residual PBAT in the composts was analyzed by chloroform extraction, an inverse correlation between the number of particles > 0.01 mm and the total extracted amount was seen, arguing for breakdown into smaller particles, but not necessarily a mass reduction. PBAT oligomers and monomers as possible substrates for subsequent biodegradation were not found. Remaining microplastic will enter the environment with the composts, where its subsequent degradability depends on the local conditions and is to date largely uninvestigated.

## Introduction

In recent years the entry of plastic^1,2^ and microplastic^1,3–12^ materials into terrestrial ecosystems has increasingly become a concern. Especially microplastic contamination can have significant negative effects on terrestrial micro- and macrofauna^5,6,13^ and has also been shown to impede plant biomass production^14^. For convenience, microplastic is often divided into large microplastic (1–5 mm) and small microplastic (< 1 mm). Among other sources, compost has been identified as potential means of entry for microplastic into the environment including arable soils^1,4,7,12,15–17^.

Compost is a popular alternative to artificial fertilizer, in particular for organic farming. Most of the above-cited studies regarding the possible contamination of composts with plastic residues tend to focus on particles and fragments > 1 mm, i.e. particles and fragments that are of relevance for current legislation, such as the regulatory restrictions for quality composts in Germany, which is given in the DüMV (Düngemittelverordnung). The DüMV specifies an upper limit of 0.1 dry wt% of plastic in the final composts but takes only particles > 1 mm into account. The regulation from the European union is less strict and allows up to 0.3 dry wt% for a given type of contaminant including plastic and considers particles > 2 mm, while the total sum of these impurities may not surpass 0.5 dry wt%^18^. Among the first to investigate the plastic contamination of different organic quality fertilizers produced in commercial or municipal composting plants with fragments > 1 mm were Weithmann et al.^16^. These authors used IR-spectroscopy to positively identify the chemical nature of the retained fragments. Composts from composting plants treating biowaste showed the highest plastic contamination (with particles > 1 mm) even though said contamination was still well within the regulatory limits^16^. In subsequent studies, simple biowaste composting plants and state-of-the-art two-stage biowaste treatment plants, where an initial anaerobic digestion step is followed by a second composting step, were investigated by the same group, with composts from the two-stage plants generally showing a more pronounced plastic contamination than those from the simple biowaste composters^1,12^. Moreover, only in composts from the investigated two-stage plants fragments > 1 mm with signatures of biodegradable plastics were found. In most cases, the recorded signatures corresponded to poly(butylene adipate co-terephthalate), PBAT, and poly(lactic acid), PLA. Therefore, it was assumed that these fragments had initially entered the biowaste treatment plants in the form of bags certified as biodegradable and sold for the purpose of biowaste collection in supermarkets close by, as for these bags PBAT and PLA were verified as main components. Such bags are typically certified according to DIN EN 13432 and 14995 which define that within 12 weeks 90% of the biodegradable bag must be disintegrated to particles < 2 mm under conditions of technical composting.

Particles and fragments > 1 mm have the advantage that they can still be identified with the naked eye. However, any of these composts may also contain particles and fragments < 1 mm, i.e. small microplastic (MP). Moreover, even plastic particles > 1 mm, after entry into the environment, may become a source of small MP, either after further break-down into several MP fragments or because of additional decay, e.g., by biodegradation. The produced MP and submicron particles may have an even more adverse effects, since the number of particles and also their surface area available for interaction with the environment will increase as larger particles break down. The likelihood for uptake by cells and tissues also increases as particles get smaller, thereby increasing the possibility of negativec effects^13,19,20^.

The systematic study of contamination of composts and in consequence the environment with small MP has been severely hampered by a lack of robust analytical methods for the detection, quantification and specific identification of particles < 1 mm in complex matrices such as compost^4,7^. To some extent biodegradable plastics such as PBAT and PLA constitute an exception in this regard, as they dissolve in chloroform. When composts suspected of containing biodegradable plastic fragments < 1 mm were extracted with chloroform, up to 0.43 dry wt% of PBAT/PLA was found^12^. However, an extraction only allows to quantify the integral of the plastic contamination, no distinction by size is possible. Only a few studies to date have studied the occurrence of microplastic particles < 1 mm type in composts. In one study plastic particles down to a size of 50 µm were considered^21^ and 2400 ± 358 such particles were found per kg dry weight. For this study, the compost was dried at 60 °C and treated with H_2_O_2_. After the digestion of organic matter, a vacuum filtration followed by a density separation was applied and approximately 20% of the retained particles suspected as being plastic MPs were analyzed by µ-FTIR. Most particles were identified as polyesters, followed by particles with signatures of PP and PE. Another study^3^ investigated particles > 30 µm and reportedly found 2800 ± 616 particles per kg of dry weight in biowaste composts and 1253 ± 561 particles per kg dry weight in greenery composts prepared from cuttings. For the analysis, adapted from Zhang et al.^22^, the particles were isolated by flotation after soil aggregates had been broken in an ultrasonic bath. The supernatant was filtered, and the particles were identified and quantified via the ImageJ software and FTIR. Only PE and PP spectra were looked for. Braun et al.^7^ isolated small and large microplastic from different composts by density separation after a treatment in an ultrasound bath to destroy aggregates and remove impurities. Particles were identified as plastic by their color, shape, and elasticity. The polymer type was not identified, neither was the lower size limit of the investigated small microplastic given. The authors found only a few small microplastic particles (ca 10 items per kg dry weight) in the composts and no significant difference between composts prepared from greenery and cutting vs composts from biowaste. They concluded that small microplastic contributes only minimally to the plastic contamination of most composts. In their review on analytical methods Bläsing and Amelung^4^ cite a few studies of small microplastic in various matrices, but none for compost. The authors proceed to include some original data on microplastic in composts but stated that “plastic fragments in the micrometer and nanometer range have not been considered here”. In their investigation of the dynamics of macro- and microplastic development during composting, Zafiu et al. recovered plastic fragment down to 200 µm from composts by sieving. Suspected particles were picked under the microscope and analyzed by FTIR. On average, the authors found 100 plastic fragments per kg of dry compost, with roughly two thirds of these in the range between 0.2 and 2.0 mm. The polymer type was indicated as a sum parameter over all fragment sizes as well as specifically for the fragments > 10 mm. PE and PP were the most frequently found polymer types in most cases. No clear development with time could be observed. Whereas biodegradable plastics were not indicated as contributing to the total number of fragments found, biodegradables made up 5% of the particles in case of the fragments > 10 mm.

The need to look at fragments < 1 mm in complex matrices such as composts has also been substantiated by a recent study from our group, where the liquid fertilizers produced by municipal two-stage biowaste treatment plants was found to contain up to 20,000 particles > 10 µm per l^1,12^. Liquid fertilizer is mainly of water, and the contained particles can therefore be analyzed by established methods for aqueous environmental samples^12^. A corresponding reliable method for the analysis of such small particles in solid digestates of composts was still missing. Here we propose such a method for the analysis of MP including the polymer type down to 10 µm in composts. All particles contained in a representative aliquot of the composts were analyzed by a suitable IR spectroscopy method. As a first demonstration of its potential, the method was used to analyze number and polymer type of fragments sizes 0.01–1.0 mm in composts from eight municipal compost producing plants: three simple biowaste composters, four plants processing greenery and cuttings and one two-stage biowaste digester-composter.

## Materials and methods

### Materials

If not otherwise indicated, the suppliers for chemicals were Th. Geyer (Renningen, Germany) and Sigma Aldrich (Taufkirchen, Germany). Ultrapure water was produced with an Elga-Veolia-Purelab (Flex2) unit, while ‘Millipore water’ came from a Millipore-Synergy-UV-system (Type 1). Protease A-01 (activity: > 1100 U mL^−1^), Pektinase L-40 (exo-PGA activity: > 900 U mL^−1^, endo-PGA activity, > 3000 U mL^−1^, pectinesterase activity: > 300 U mL^−1^), and Cellulase TXL (activity: > 30 U mL^−1^) were obtained from ASA Spezialenzyme GmbH (Wolfenbüttel, Germany). Viscozyme L (activity: > 100 FBG U g^−1^) was obtained from Novozymes A/S (Bagvaerd, Denmark).

### Investigated composting plants

Composts produced by eight different composting plants all located in Southern Germany were included in this investigation (Table 1). The plants were chosen with the aim of keeping the total number manageable, while covering current state-of-the-art technology for the production of quality composts from either greenery and cuttings or biowaste. The plants receive a mix of material of rural and suburban provenance. None of the plants processed material from a metropolitan area. Three of these plants (identified below as plants #1 to #3) produced compost from municipal biowaste together with added cuttings for structuring, plants #4 to #7 produced their compost exclusively from greenery and cuttings. Plant #8 is a biowaste processing plant with a fermentation step followed by composting of the solid digestate. For this the original digestate is separated into liquid and solid digestate using a screw extruder. All plants produce a certified high-quality compost suitable for use in agriculture and gardening. Samples were taken from the finished compost., i.e., the material obtained at the end of the process including the final sieving step. This final sieving step has previously been identified as quite effective to remove fragments > 5 mm^1^. The removal of smaller particles by sieving poses more of a challenge. Increasingly finer sieves would be needed and more of the desired organic material would be removed.

**Table 1:** Technical details of the investigated plants. Plants #1 to #3 produce compost from biowaste, plants #4 to #7 from greenery and cuttings, plant #8 is a two-stage system for biowaste treatment first by anaerobic digestion followed second by composting of the solid digestate.

Plant number	Substrate preparation	Fermentation	Composting	Final sieving (mm)
#1	Bag slicer, sieving (80 mm); magnetic separator	–	12 weeks	10–15
#2	Sieving (80 mm)	–	10 weeks	12
#3	Bag slicer, sieving; (80 mm)	–	6–7 weeks	10
#4	Shredder	–	Composting to a degree of rotting 3–5	10
#5	Shredder	–	3–4 months	15
#6	Shredder	–	3–4 months	15
#7	Shredder	–	6 weeks	20
#8	Shredder; sieving (60 mm)	Fermentation ~ 21 days, then separation of liquid and solid digestate	6 weeks	10

### Sampling of composts

Sampling of compost was done like described in previous studies^1,12^. Bulk samples were taken from the composts according to the guidelines of the German Association for Quality Compost^23^. A slight modification to the standard procedure was introduced to avoid additional contamination of the compost samples with plastics, particularly via the plastic foil floor cover recommended in the standard protocol for sample mixing. Instead, the individual aliquots obtained from a given compost heap were pooled, mixed and stratified directly on the concrete floor (after a ‘washing’ step with compost from the same heap). To obtain a representative sample, the interior of the heap was made accessible by wheel loader. Then, individual samples were taken at evenly spaced points. The number and volume of individual samples depended on the volume and grain size of the compost pile, as prescribed by the guidelines. Typically, for the composts considered here (grain sizes “12–20 mm”) assuming a 100 m^3^ compost heap, 16 individual samples (1 L each) were taken, and a minimum of 4 mixed samples (2 L each) were created.

To avoid contamination of the samples with plastic fragments from the ambient air, clothing, laboratory tools, or reagents used during sample processing, precautionary measures were taken as previously described^1,12^. Cotton lab coats were worn throughout. Unless direct access was necessary, samples were covered with glass or aluminum foil lids. Sample processing took place in a laminar-flow box to prevent airborne particles from contaminating the samples. All laboratory tools used were made of glass, metal, or polytetrafluorethylene (PTFE), a polymer that is rarely found in environmental samples and in addition excluded here from the analysis. All required solutions and the deionized water used were filtered through 0.2 μm pore membranes (mixed cellulose ester membrane, diameter 47 mm, Whatman ME 24, Merck KGaA) before use. Enzyme solutions were filtered through 0.45 μm pore membranes (regenerated cellulose membrane, diameter 100 mm, Whatman RC 55, Merck KGaA) and stored in glass bottles with glass caps until use. All laboratory equipment was thoroughly rinsed with filtered deionized water, 35% ethanol, and again with filtered water before use and between steps to avoid cross contamination. Blanks subjected to the same treatment as the environmental samples were used to detect possible contamination in the laboratory.

### Analysis of plastic fragments > 1 mm in the composts and determination of the compost dry weight

In preparation for analysis, the compost samples were filled into a rectangular metal form (790 mm × 510 mm × 150 mm), thoroughly mixed with a metal shovel, and quartered. Samples were taken from two quarters (bottom right and top left) for analysis of the plastic content. Samples for the determination of the compost’s dry weight were taken from the bottom left quarter, while backup samples (1 L) were taken from the top right quarter. For the determination of the dry weight, 100 mL sample aliquots were weighed into 250 mL Schott-Duran beakers and dried at 105 °C (oven: Memmert UM 500, Memmert, Schwabach, Germany) for at least 24 h. Afterward, the beakers were allowed to cool to room temperature in a desiccator, and the dry weight was determined by reweighing the beakers.

For analysis of fragments > 1 mm, approximately 3 L of compost sample was weighed and evenly distributed into 6 glass vessels (capacity 3 L each). The material was suspended in 2.5 L of water and sieved with a mesh size of 1 mm, followed by collection of the retained particles (fraction > 1 mm). The sieve was obtained from Retsch GmbH (Haan, Germany; test sieve, IS 3310-1; body/mesh, S-steel; body, 200 mm × 50 mm). For the analysis of the chemical nature of the particles, attenuated total reflection-Fourier transform infrared (ATR-FTIR) spectroscopy (spectrometer: Alpha ATR unit, Bruker, equipped with a diamond crystal for measurements) was used. Spectra were taken in the wavenumber range from 4000 to 400 cm^−1^ (resolution 8 cm^−1^, 16 accumulated scans, OPUS 7.5 software) and compared with entries from an in-house database described previously^24^ or the database provided by the manufacturer of the instrument (Bruker). An incident light microscope (microscope, Nikon SMZ 754 T; digital camera, DS-Fi2; camera control unit, DS-U3; software, NIS Elements D) was used for visual documentation and dimensional analysis of all particles identified by ATR-FTIR as synthetic plastics.

### Extraction and quantification of residual PBAT/PLA as bulk from compost samples

Putative residual PBAT and PLA was extracted in bulk from compost samples after removal of fragments > 1 mm using a previously published method^12,25^ in modified form. Compost aliquots were first sieved through a 1 mm mesh as described above to remove the larger fragments, and then dried at 60 °C for 48 h prior to extraction. One hundred grams of the dried material was placed in 500 mL glass bottles and 250 mL of a 90/10 vol% chloroform/methanol mixture was added. The glass bottles were sealed, placed on a horizontal shaker for 10 min and subsequently sonicated in a water bath at room temperature for 10 min. Afterwards, the containers were placed overnight in a fume hood. The next day, the contents were passed through a Büchner funnel under vacuum and the retained residues were washed with excess chloroform to remove any remaining dissolved material. The solvents were removed from the filtrate by rotary evaporation under vacuum and the obtained residue was dried overnight in an oven at 45 °C under vacuum. To quantify polymer content and composition, ^1^H-NMR spectra were recorded in CDCl_3_ for each extract. As indicated either 1,2-dichloroethane or methanol was chosen as inner standard, as both substances generate peaks in areas where they do not interfere with the peaks of PLA and PBAT. The peak intensity of ^1^H-NMR is proportional to the number of protons in the molecule. Taking this into account, the integration values of peaks were used for quantification of the analyte molecules. The amounts of PBAT and PLA calculated for the extracts were then normalized to the dry weight of the extracted compost sample and used for the calculation of the total mass concentration (wt%) of PBAT and PLA per unit of dried compost.

To validate the recovery of PBAT and PLA from compost via extraction, a greenery compost from the Bayreuth University’s Botanical Garden verified to contain no PBAT/PLA was taken and dried. 100 g aliquots of this dried compost were spiked with respectively 500 ppm, 1500 ppm, 3000 ppm and 5000 ppm of PBAT and PLA and mixed well. The prepared composts were then extracted, and the extracts analyzed as described above. Aliquots from the compost without spike served as negative control. All experiments were done in triplicate.

### Analysis of plastic fragments 0.01-1.0 mm in the composts

To isolate microplastic particles in the range of 0.01-1.0 mm from the composts, three subsamples of 10 g were taken from each of the thoroughly mixed compost samples. Using an adapted protocol inspired by Scopetani et al.^26^, the subsamples were mixed with pre-filtered (0.2 µm) deionized water and wet-sieved through a 500 µm stainless steel mesh. The residue was visually analyzed for retained particles, any found fragments were analyzed by ATR-FTIR. The filtrate was collected in a clean glass beaker and filtered over a 10 µm stainless steel filter (47 mm diameter, Rolf Körner GmbH) with a vacuum filtration unit (3-branch stainless steel vacuum manifold with 500 mL funnels and lids, Sartorius AG). The filter cake was then mixed with 50 mL of a sodium dodecyl sulfate solution (≥ 95% SDS; Karl Roth) for 24 h at room temperature, which was subsequently filtered again over the 10 µm filter. The filter cake was then treated with 40 mL Fenton’s reagent (20 mL of 30% H_2_O_2_ and 20 mL 0.05 M Fe(II) solution (composed of 7.5 g iron(II) sulfate heptahydrate (FeSO_4_⋅7H_2_O) in 500 mL ultrapure water and 3 mL concentrated sulfuric acid (H_2_SO_4_)). Subsequently, the Fenton’s reagent was filtered off (10 µm filter) and the filter cake was washed into a polytetrafluorethylene (PTFE) tube (10 cm long, 3 cm inner diameter) with 30 mL filtered deionized water. Then 5 mL rape seed oil were added, and the tube was closed with a rubber stopper. The tube was thoroughly shaken to allow the oil to get into contact with the sample. After two hours of settling time, the tubes were put into a − 20 °C freezer overnight to solidify. The frozen water–oil column was then pushed out of the PTFE tube, where the oil fraction containing the particles could easily be separated from the ice-column, that entraps the compost residues. The oil was then allowed to melt on a 10 µm filter and was filtered off. The filter was rinsed again with the SDS solution to remove residual oil. Subsequently, ¼ of the purified sample was transferred onto aluminum oxide filters (0.2 μm, Anodisc, Whatman GE Healthcare) to then be analyzed by µ-FTIR (Bruker Hyperion 3000 FTIR microscope (Bruker), equipped with a 64 × 64-pixel FPA detector in conjunction with a Tensor 27 spectrometer). The samples were measured in the transmission mode with a 3.8 × IR objective (spatial resolution 11.05 µm) and a wave number range of 3600–1250 cm^−1^ with a resolution of 8 cm^−1^ and a coaddition of 6 scans. Data processing was conducted using the Bruker OPUS software version 7.5 (Bruker Optik GmbH).

## Results and discussion

### MP contamination in the investigated composts

Compost from three different types of composting plants, see Table 1 for details, were examined by the methods described in the experimental section. The focus of this study was on particles < 1 mm, since with the exception of plant #8, data on particles > 1 mm found in these plants have recently been published^1^. According to this previous investigation, the simple biowaste composts (plants #1 to #3) contained on the average 13.5 plastic fragments > 1 mm per kg of dry compost (standard deviation 2.23), while the greenery & cuttings composts (plants #4 to #7) contained 20 such fragments per kg, albeit with a large standard deviation of 17.85. None of these composts contained fragments > 1 mm with a signature of a biodegradable polymer such as PBAT. By comparison, the compost from the biowaste digester-composter (plant #8), investigated here for the first time, contained 260 fragments > 1 mm per kg of dry compost. Most of these fragments (225) had a signature of PE, 30.5 a signature of PP. Other polymer types were found, if at all, in single digit numbers. The results obtained for plant #8 are thus in the same order-of-magnitude as those previously published by us for similar two stage biowaste treatment plants^1^. However, contrarily to most of the two-stage biowaste treatment plants investigated before, the compost from plant #8 contained no fragments > 1 mm with a PBAT signature.

Table 2 summarizes the number and type of the plastic particles < 1 mm per kg dry weight found in the investigated composts, while Tables 3, 4 and 5 show the size distribution of the plastic particles < 1 mm averaged over the respective plant types. The majority of the small microplastic fragments is found in the 22–100 µm range. This is the case for all investigated plastic types and plant technologies. Hardly any particles could be detected in the range > 300 µm. The reason for the conversion into that particular size fraction is not quite clear, however, it is possible that mechanical stress, in particular shear stress, becomes less and less important as degradative influence as the particles get smaller. In their investigation on the dynamics of the microplastic contamination during technical composting Zafui et al. showed how mechanical stress influences breakdown of microplastic into smaller fragments^15^. These authors also observed a trend towards the formation of particles in the size range 0.63–0.2 mm. Unfortunately, particles < 0.2 mm were not investigated by these authors.

**Table 2:** Microplastic fragments/particles 0.01-1.0 mm found per kg of dry compost. Plants #1 to #3 produce compost from biowaste, plants #4 to #7 from greenery and cuttings, plant #8 is a two-stage system for biowaste treatment first by anaerobic digestion followed second by composting of the solid digestate, three technical triplicates each.

Plant number	Biowaste					Greenery & cuttings						Biowaste
	#1	#2	#3	Average	SD	#4	#5	#6	#7	Average	SD	#8
PBAT	5123	1570	2884	3192	1797	0	0	0	0	0	0	2079
PP	603	1570	384	852	631	649	703	3460	1110	1480	1336	331
PE	301	897	1153	784	437	162	0	546	888	399	398	378
PS	0	0	0	0	0	324	0	0	444	192	227	898
PET	0	224	0	75	129	0	234	0	0	59	117	47
PVC	0	0	192	64	111	0	234	0	0	59	117	47
Other particles	0	448	384	278	243	0	468	364	0	208	244	0
Sum	6027	4709	4998	5245	693	1135	1640	4371	2442	2397	1422	3780

*SD* Standard deviation.

n = 9 for simple biowaste composts, n = 12 for composts from greenery & cuttings.

**Table 3:** Average percentage of the polymer types in different size fractions found in the composts from the three simple biowaste composting plants (#1-#3).

	11–22 µm	22–100 µm	100–300 µm	300–500 µm	Sum
PBAT	16.19%	39.62%	5.05%		
PP		13.60%	2.65%		
PE	2.85%	8.43%	3.67%		
PS					
PET		1.43%			
PVC	1.22%				
Others	1.43%	3.87%			
Sum	21.69%	66.95%	11.36%

**Table 4:** Average percentage of the polymer types in different size fractions found in the composts from the four plants composting greenery & cuttings (#4-#7).

	11–22 µm	22–100 µm	100–300 µm	300–500 µm	Sum
PBAT					
PP	3.80%	49.41%	8.56%		
PE		12.65%	4.01%		
PS		8.01%			
PET		2.44%			
PVC	2.44%				
Others		4.89%	3.80%		
Sum	6.24%	77.39%	16.36%

**Table 5:** Percentage of the polymer types in different size fractions found in the composts from the two-stage biowaste digester-composter (#8).

	11–22 µm	22–100 µm	100–300 µm	300–500 µm	Sum
PBAT	2.50%	41.25%	10.00%	1.25%	
PP		2.50%	5.00%	1.25%	
PE		2.50%	6.25%	1.25%	
PS		12.50%	11.25%		
PET			1.25%		
PVC			1.25%		
Others					
Sum	2.50%	58.75%	35.00%	3.75%	

### Effects of the compost type on microplastic contamination with conventional commodity plastic

The contamination of composts with microplastic in the size range of 0.01–1.0 mm is investigated here in detail for the first time. Rather than mirroring the situation documented for particles > 1 mm, significant differences are observed. In case of fragments and particles > 1 mm the average contamination with commodity plastic residues, such as PE and PP, had been similar for quality composts prepared for biowaste and greenery & cuttings, while that of the composts from various two-stage biowaste treatment plants had been roughly an order of magnitude higher^12^. According to the data presented here, the contamination of the compost from the two-stage plant with microplastic fragments/particles in the size range of 0.01–1.0 mm was in the same range as that of the other investigated composts. Moreover, whereas PE, which is the preferred polymer for bags and wrappings, tended to dominate amongst the plastic fragments > 1 mm^1,12^, this is apparently not the case for the small microplastic fraction. With the exception of that from plant #3, all investigated composts, biowaste and greenery composts alike, contained more fragments/particles with a PP than with a PE signature.

Table 6 summarizes the ratio between the number of microplastic particles (0.01–1.0 mm) to the number of particles > 1 mm found in the composts for PE respectively PP. In most cases the ratios are higher for PP than for PE. This together with the fact mentioned above that PP rather than PE tended to be the dominant small microplastic type amongst the commodity plastics in the composts suggests a possibility that PP has a higher tendency to disintegrate into microplastic than PE. This is corroborated by the data from plant #2, where no ratio could be calculated for PP, since no PP particles > 1 mm were found, but where the corresponding compost contained the highest amount of small PP microplastic among the compost of the simple biowaste composters , as weel as by plant #5 where PE fragments were only found in the > 1 mm fraction, but not at all in the microplastic fraction. A trend of more pronounced fragmentation of PP compared to PE has also been observed in other studies^27^. Several factors can contribute to this. It is known that that as a polymer PP degrades faster than, e.g., LDPE if the polymers are exposed to the same environmental conditions like oxidation and are similar in material properties such as the crystallinity^28^. Under these circumstances, PP is more prone to degradation than PE, since the hydrogen in PP can form radicals more easily. The positive inductive effect of the additional methyl group in PP, helps to stabilize the tertiary carbon atom. Subsequently, the addition of oxygen is facilitated, which in turn prevents the formation of unstable secondary products and further radicals. Thus, an autocatalytic chain reaction is started. However, while this is the case for the polymers, in case of actual plastic products, other factors such as additives (in particular antioxidants as often added to PP), stabilizers, and fillers, but also the source (e.g. rigid product vs packaging foil) will also influence stability. Whether this had an effect here is difficult to ascertain. Morphological information would have been useful in this context, but this is difficult for fragments/particles < 500 µm.

**Table 6:** Ratios of PE and PP microplastic particles to particles > 1 mm of the same polymer type.

Plant number	Biowaste					Greenery & cuttings						All composts		Biowaste
	#1	#2	#3	Average	SD	#4	#5	#6	#7	Average	SD	Average	SD	#8
PP	490		166	328	229	108		2703	178	996	1479	729	1114	11
PE	35	196	167	133	86	67		213	30	109	97	118	83	2

When simple biowaste composts are compared to composts from greenery and cuttings, the contamination with small PP microplastic compared to small microplastic from PE is even more pronounced for the greenery and cuttings compost. Two effects conceivably contribute to this. First, the data on the contamination with fragments > 1 mm also show a higher contamination of the greenery and cuttings composts with these commodity plastics, suggesting that perhaps more PE and PP enters or remains in the respective process stream, either due to a more contaminated input material or less effective removal of contaminants from the input material. Typically, composting plants take much more stringent measures to improve the quality of the incoming biowaste than of the greenery and cuttings entering the plant. Together with the presumed higher tendency for disintegration of PP compared to PE, this could conceivably result in the observed higher contamination of the compost from greenery and cuttings with PE and PP microplastic.

### Effects of the compost type on microplastic contamination with the biodegradable plastic PBAT

The most striking difference between the different compost types was seen in case of the contamination with PBAT, typically considered a “biodegradable” material^29^. This was even more surprising, since no PBAT fragments > 1 mm had been found in any of the investigated composts, including the compost from plant #8. In the small microplastic range investigated here, the investigated greenery & cuttings composts contained no small PBAT microplastic, arguing that indeed no such material entered the process stream. In contradistinction, all biowaste composts contained a significant number of small microplastic particles/fragments with a PBAT signature. In fact, often the largest fraction of the small microplastic particles found in the biowaste composts (e.g., > 80% in case of plant #1), had a PBAT signature and this regardless of whether the composts had been prepared by simple composting or by the two-stage digestion-composting process in plant #8. Moreover, the simple biowaste composting plants, which had consistently shown no contamination with PBAT fragments > 1 mm in our previous investigation^1^, tended to have a higher contamination with small PBAT microplastic fragments than the compost produced in plant #8. In terms of further breakdown, PBAT shows a similar behavior as discussed above for the conventional commodity plastics. Over 80% of the PBAT fragments were found in the 22–100 µm fraction, i.e., the biodegradable PBAT showed no pronounced higher tendency to break down into smaller particles than the conventional plastic.

### Effects of the plant technology on PBAT microplastic contamination

Several factors can be expected to contribute to the breakdown of PBAT-based materials in the investigated plants, including: (1) The quality of the incoming material; (2) the duration and intensity of the composting phase, and (3) the process technology of the plants, in particular the initial treatment of the incoming biowaste and that of the final compost. The quality of the incoming material was not investigated in this study, which focusses on the quality of the final composts. So, this aspect is not taken into consideration here. It has, e.g., been studies by Zafiu et al. in their study of the dynamics of microplastic development during composting^15^. For the breakdown of biodegradable materials, the length and perhaps also the intensity of the composting phase is commonly considered to be of high importance. In our study, no such correlation is observed. With 6.5 weeks the composting phase is shortest in plant #3, where the PBAT contamination is twice as high as in plant #2 (10 weeks), albeit only half that of plant #1 (12 weeks). However, the observed differences may be related to the quality of the input material, which was not known to us, see above.

Regarding technology, all biowaste treatment plants included in this study pass the incoming material through a sieve (either 80 or 60 mm, for details see Table 1). At plants #1 and #3 a bag slicer is in addition installed. The final compost is sieved with meshes of 10–15 mm, 12 mm and 10 mm respectively in plants #1 to #3. The plants treating greeneries & cutting (#4 to #7) use only a shredder to prepare the incoming material, while in plant #8 the material is shredded and then sieved using a 60 mm mesh. After the digestion step in plant #8, the liquid and solid digestate are separated via screw press. This may promote plastic fragmentation due to the mechanical forces of the pressing process. The final compost is sieved with a 10 mm mesh in plant #8.

Composts from plants using a bag slicer, i.e., in particular those produced in plants #1 and #3, contain a larger number of small microplastic than any of the others, including biowaste composts produced in plants #2 and #8. However, this is largely due to the high amount of PBAT microplastic found in these plants. It is possible that the initial effect of the bag slicer is similar in all cases, i.e., the production of small plastic fragments in the mm-range. However, while these fragments then remain fairly large in size in case of the commodity plastics, hence a reduction of their number by the final sieving step remains possible, in case of PBAT particles of simmilar size break down into fragments < 500 µm. Particles of this size cannot be removed by the sieving step; hence the fairly large numbers of small PBAT microplastic fragments that are found in the corresponding composts. Any PBAT-based microplastic still present in the composts at the end of the process will enter the environment, when the compost is used as organic fertilizer. Little is currently known about subsequent degradation in the environments, since the certification of a plastic material as “biodegradable” focusses on conditions during technical composting. However, evidence has been presented that further break-down may be slow and the corresponding microplastic may in fact persist as such in the environment for quite some time^30^. The environmental consequences of such a contamination of composts with large amounts of small PBAT microplastic need to be assessed before the practice of releasing such composts as fertilizer for agriculture and gardening is continued.

### Chloroform extraction to assess to bulk contamination of the composts with PBAT

Finally, the bulk of the PBAT contamination of the investigated composts was investigated via chloroform extraction following a previously published protocol^12^. Chloroform dissolves PBAT and incidentally also PLA quite effectively, while this is not the case for commodity plastics such as PP and PE or any other of the synthetic plastics typically found as contaminants in the composts. However, chloroform extraction provides only data on the bulk polymer amounts, not on the presence and/or  distribution of differently sized particles.

**Figure 1:**
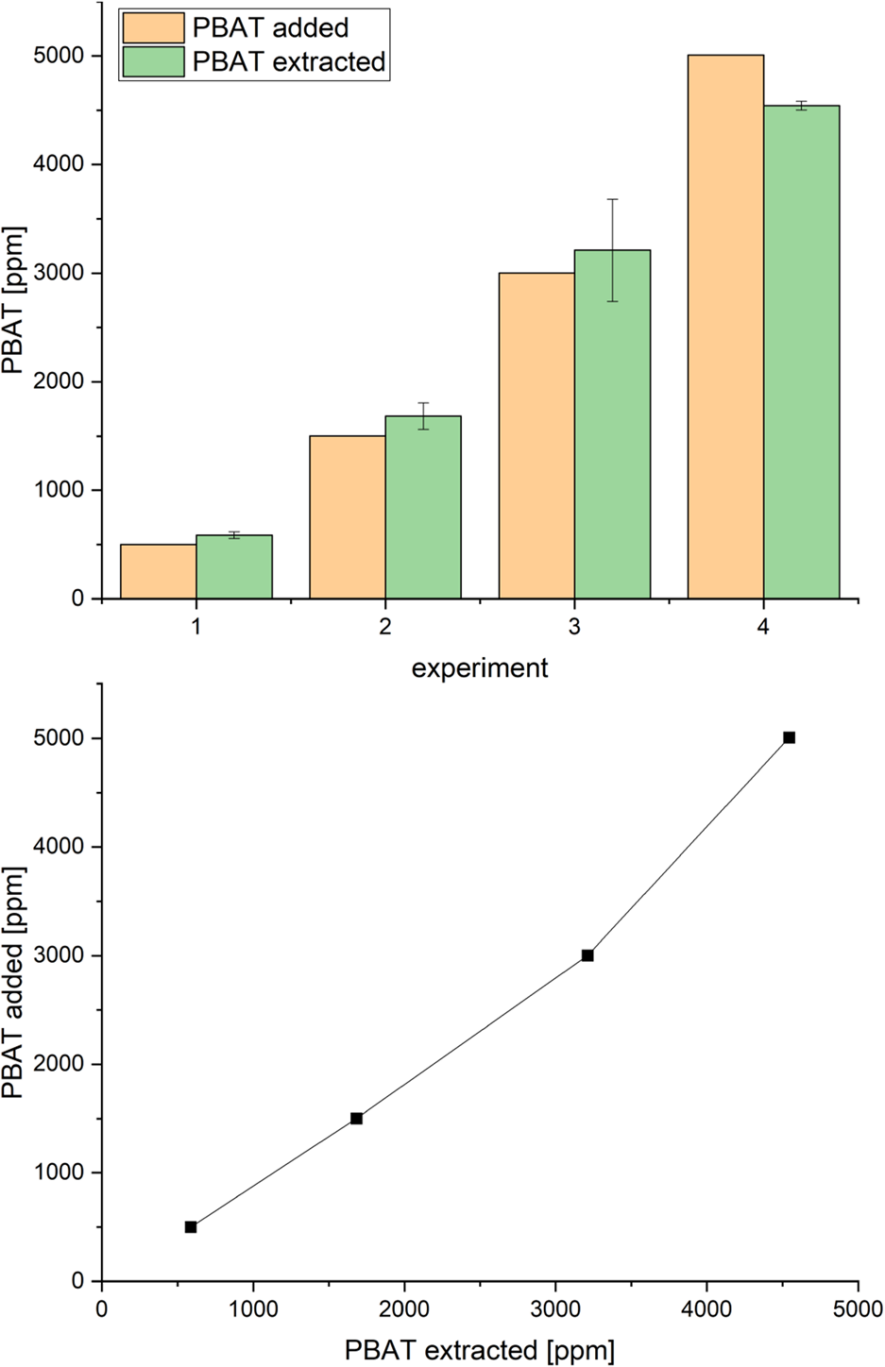
Recovery of PBAT from spiked compost samples. The error bars represent the standard deviation (n=3).

To validate the extraction procedure, composts from the University’s botanical garden were spiked with four different concentrations of PBAT and PLA. PLA was added to increase the complexity of the sample by adding another chloroform dissolvable biodegradable polymer. Beforehand it was verified that the compost from the botanical garden, a pure greenery & cutting compost, did not contain any detectable PBAT contamination. Subsequently the compost from the botanical garden served as negative control in the actual measurements. Results are shown in Fig. 1 and demonstrate that the method is applicable and suitable to determine PBAT in composts.

**Figure 2:**
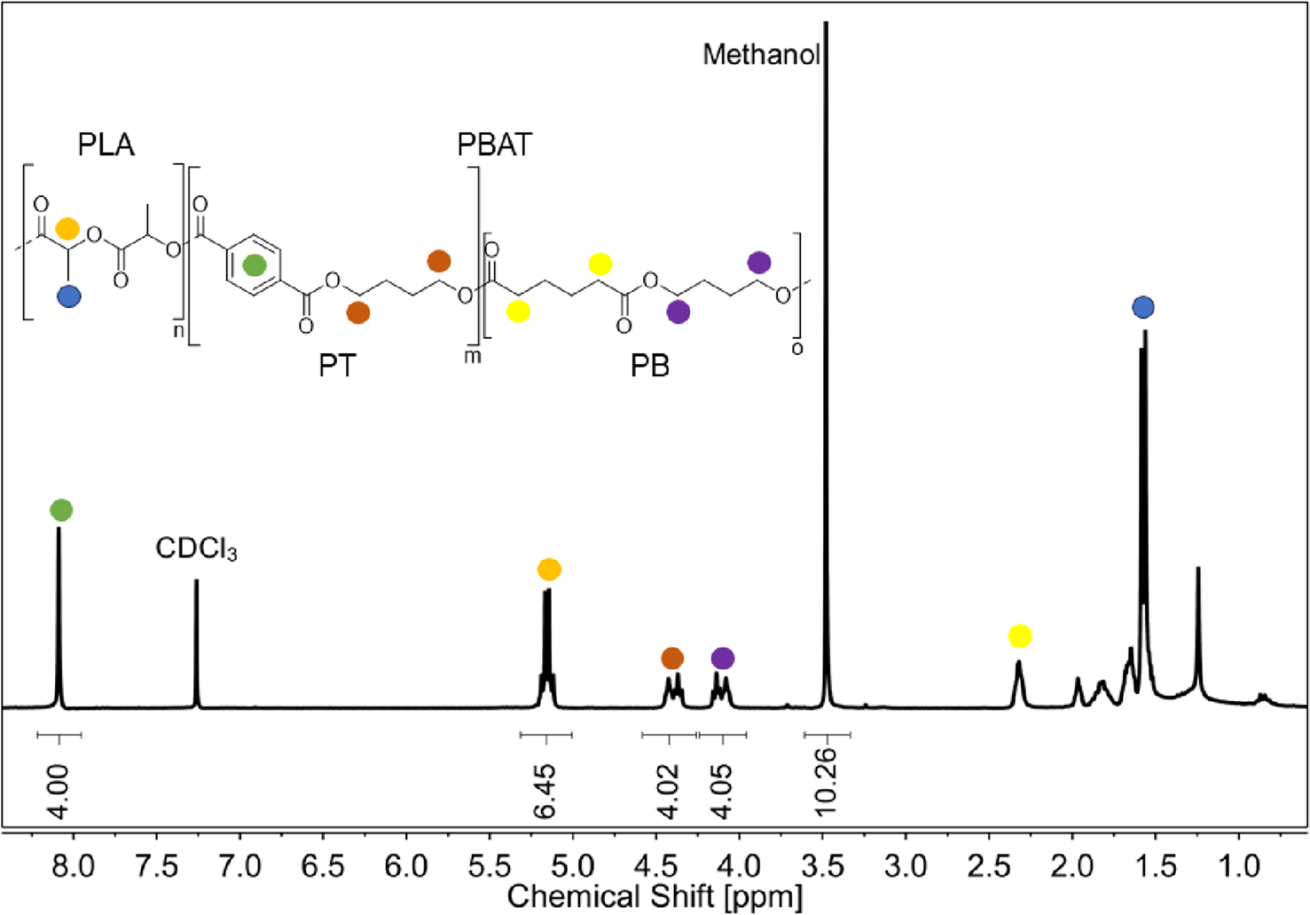
Exemplary ^1^H-NMR of the polymer material obtained via chloroform extraction from the compost samples, measured in CDCl_3_, with methanol as internal standard. The signals which were integrated for the quantification of PBAT are indicated by color-code. Values obtained via the integration are indicated below the respective peaks.

The data for the PBAT amounts determined by ^1^H-NMR in the various composts are compiled in Table 7, an exemplary ^1^H-NMR spectrum is shown in Fig. 2. The PBAT concentration in the composts was calculated as described previously^12^. Please note, while the extraction will recover all PBAT fragments still present in the composts regardless of size, we never saw any evidence for oligomeric PBAT or the corresponding monomers in the recorded spectra. In such cases signals from the end groups would be discernable, which is not the case, see Fig. 2.

**Table 7:** Mass concentrations of PBAT the in composts.

Plant number	#1	#2	#3	#4	#5	#6	#7	#8
M_c_ [g]	100	70	85	100	70	100	70	100
M_e_ [g]	2.272	1.506	0.756	0.722	0.364	1.022	0.587	0.695
C_PBAT_ [ppm]	124	153	361	n.d	n.d	n.d	n.d	965

M_c_: mass of dry compost subjected to extraction; M_e_: mass extracted from compost sample; C_PBAT_: mass concentration of PLA in the compost (M_c_); n.d.: not detectable.

None of the greenery & cuttings composts showed any detectable contamination with PBAT. Since the chloroform extraction will recover all PBAT down to the nano and molecular scale, this strongly suggests that the problem of PBAT residues is restricted to composts prepared from biowaste. The source of the PBAT in the biowaste composts can only be speculated upon since the input material was not investigated. However, PBAT is currently mainly used as alternative for PE in the preparation of foils. Therefore, it is likely that the PBAT entered the biowaste via packaging or even the PBAT-containing “biodegradable” bags promoted specifically for biowaste collection.

Biowaste composts from plant #1 to #3 contained between 124 and 361 g of PBAT per ton of dry compost. The amount of PBAT in the input material was not determined and will of course influence the residual contamination. However, there seems to be a rough correlation between the PBAT amount and the composting time. In particular for the compost from plant #3 (6–7 weeks of composting) the residual PBAT contamination is approximately twice as high as for the composts from the two other plants (plant#1 and plant #2), which use 12 and 10 weeks of composting respectively.

The highest mass concentration of PBAT, namely 965 g of PBAT per ton of dry compost, was found in the compost from plant # 8. i.e., the plant using digestion followed by a brief, 6-weeks composting step. This is surprising, since the contamination with PBAT fragments in the 0.01–1.0 mm range of this compost had been similar to or even lower than that of the biowaste composts from plants #1 to #3. Moreover, the value is also high compared to most of the previously analyzed composts from similar two stage biowaste treatment plants, where incidentally a contamination with fragments > 1 mm had been observed^12^. It seems that less contamination of compost with larger fragments of PBAT plastic corresponds to a higher contamination with smaller fragments. This is also the case for plants #1 and #3. Plant #2 deviates from this rule to some extent (low number of particles, low total mass concentration). The reasons can at present only be speculated upon. It is possible that the fact that no bag slicer is used and in consequence less mechanical stress acts on the material during processing contributes or that differences in the initial input contamination are responsible. Moreover, whether this progress towards smaller fragments/particles is due to—beneficial—biodegradation and concomitant overall mass reduction or simply caused by a—problematic—breakdown into smaller, more numerous and potentially more noxic particles will need further research. However, the large increase in the number of the smaller sized PBAT-fragments as breakdown continues argues against pronounced biomineralization. Possible environmental consequences of the increasing numbers of PBAT micro and nano plastics in compost used as fertilizers still need to be investigated.

## Conclusions

Microplastic < 1 mm, specifically fragments/particles in the range of 0.01–1.0 mm, was made accessible herein for a detailed investigation of number, size, and polymer type in compost. This opens the possibility of further detailed studies of the dynamics of microplastic development during biowaste treatment. Given the potential impact of such small microplastic on the environment, an expansion of our studies to additional plastic materials, input streams, and biowaste treatment processing types is necessary. Moreover, for the first time it has been possible to study the large and small microplastic fraction by size and polymer type down to 10 µm. This is an important prerequisite for improving both plastic materials, in particular the so-called biodegradable materials, but also the technology used, e.g. in biowaste treatment plants.

Regarding the contamination of the composts with plastic having signatures of conventional commodity plastics, it was surprising to see that PE, which tended to dominate the fraction > 1 mm was much less prevalent in the small microplastic fraction, where PP tended to dominate instead. We propose that this is at least partially due to the better chemical stability of PE. Fragments created e.g. by the bag slicer or shredder from incoming PE bags and foils tend to be in the mm-size and may show little propensity for further breakdown during composting. Fragments of that size can be effectively removed by the final sieving step. On the other hand, less PP is likely to enter the plants in the beginning, but the produced mm-fragments break down more easily during composting into the much more difficult to remove small MP fragments.

The biggest surprise was the domination of the microplastic fraction of the biowaste composts by PBAT, while no such material was ever found in the composts prepared from greenery and cuttings. PBAT is a popular and widely exploited biodegradable polymer used not only in bags for biowaste collection, but also as alternative to PE/PP for packaging and other application due to its favorable material properties^29,31–34^. Since PBAT-fragments > 1 mm were rarely found in simple biowaste composts^12^, the material is often considered unproblematic or even beneficial for biowaste collection. However, here we find large numbers of PBAT fragments < 1 mm in the simple biowaste composts. By comparison, none of the composts generated from greenery and cuttings showed any contamination with PBAT.

It seems thus likely that all biowaste composts contain residues of PBAT and that disintegration of the PBAT advances with composting times. This would explain our inability to find larger microplastic in most investigated biowaste composts. However, this disintegration into smaller particles does not necessarily lead to a reduction in the total mass of the material, since PBAT tends to dominate in the microplastic fraction and the extraction of the PBAT in bulk from the composts showed that, if anything, compost with a comparatively small number of residual discernable particles > 10 µm tended to have a higher content in total PBAT. Finally, the reduction in size does not necessarily stop at 10 µm even though most of the fragments recovered by us were between 22 and 100 µm in size.

Since the subsequent fate of any PBAT entering the environment as well as the environmental consequences of such an entry are not clear at present, the avoidance of any plastic contamination in organic waste should remain a priority. Further research is necessary at all levels from the processes at the plants to the effects of composted plastics in the environment and their further degradability, but also the effects of a putative accumulation of aged microplastics from compost in the soil, before PBAT is unreservedly promoted as environmentally friendly alternative to commodity plastics such as PE.

## Data Availability

All data are available in the main text.
